# Characterization of CTX-M ESBLs in *Enterobacter cloacae*, *Escherichia coli *and *Klebsiella pneumoniae *clinical isolates from Cairo, Egypt

**DOI:** 10.1186/1471-2334-9-84

**Published:** 2009-06-04

**Authors:** Noha G Khalaf, Mona M Eletreby, Nancy D Hanson

**Affiliations:** 1 Department of Microbiology and Immunology, Faculty of Pharmacy Helwan University, Helwan, Egypt; 2 Clinical Microbiology department, National Heart Institute, Cairo, Egypt; 3 Center for Research in Anti-Infectives and Biotechnology, Department of Medical Microbiology and Immunology, Creighton University School of Medicine, Omaha, Nebraska, USA

## Abstract

**Background:**

A high rate of resistance to 3^rd ^generation cephalosporins among Enterobacteriaceae isolates from Egypt has been previously reported. This study aims to characterize the resistance mechanism (s) to extended spectrum cephalosporins among resistant clinical isolates at a medical institute in Cairo, Egypt.

**Methods:**

Nonconsecutive *Klebsiella pneumoniae *(Kp), *Enterobacter cloacae *(ENT) and *Escherichia coli *(EC) isolates were obtained from the clinical laboratory at the medical institute. Antibiotic susceptibility was tested by CLSI disk diffusion and ESBL confirmatory tests. MICs were determined using broth microdilution. Isoelectric focusing (IEF) was used to determine the pI values, inhibitor profiles, and cefotaxime (CTX) hydrolysis by the β-lactamases. PCR and sequencing were performed using *bla*_CTX-M _and IS*Ecp1*-specific primers, with DNA obtained from the clinical isolates. Conjugation experiments were done to determine the mobility of *bla*_CTX-M_.

**Results:**

All five clinical isolates were resistant to CTX, and were positive for ESBL screening. IEF revealed multiple β-lactamases produced by each isolate, including a β-lactamase with a pI of 8.0 in Kp and ENT and a β-lactamase with a pI of 9.0 in EC. Both β-lactamases were inhibited by clavulanic acid and hydrolyzed CTX. PCR and sequence analysis identified *bla*_CTX-M-14 _in Kp and ENT and a *bla*_CTX-M-15 _in EC. Both *bla*_CTX-M-14 _and *bla*_CTX-M-15 _were preceded by IS*Ecp1 *elements as revealed by partial sequence analysis of the upstream region of the *bla*_CTX-M _genes. *bla*_CTX-M-15_ was transferable but not *bla*_CTX-M-14_.

**Conclusion:**

This is the first report of CTX-M-14 in Kp and ENT isolates from Egypt, the Middle East and North Africa.

## Background

Recent studies on Enterobacteriaceae isolates from Egypt have reported a resistance rate to third generation cephalosporins of 70% [1,2]. A survey, carried out in 2001–2002 and covered medical centers in Northern and Southern European countries, Egypt, Lebanon, Saudi Arabia and South Africa, reported the highest incidence of extended spectrum β-lactamases (ESBLs)-producing isolates in Egypt [3].

CTX-M ESBLs are the most prevalent ESBLs worldwide [4]. Recently, CTX-M ESBLs have been reported in Egypt [5], with CTX-M-15 being the most common ESBL reported in the Middle East region and North Africa [6,5,7]. However, CTX-M-14 has also been detected in *Escherichia coli *isolates from Egypt and Tunisia [5,8]. But CTX-M-14 has not been reported in *Klebsiella pneumoniae *isolates in this geographical region before.

CTX-Ms are class A ESBLs that are most active against cefotaxime [9]. However, some CTX-Ms can hydrolyze ceftazidime such as CTX-M-15 and CTX-M-19 [10,11]. The nucleotide sequences of *bla*_CTX-M _genes are highly related to the nucleotide sequence of *Kluyvera spp. *[12,13].

Clinical isolates of *K. pneumoniae*, *Enterobacter cloacae *and *E. coli *were sent from the clinical microbiology laboratory in a medical institute in Cairo, Egypt to investigate the mechanism (s) responsible for resistance to extended spectrum cephalosporins.

## Methods

### Bacterial strains

Five clinical isolates were sent on blood agar plates from the clinical laboratory at the medical institute. The isolates were three nonconsecutive *K. pneumoniae *isolates and one *E. coli *isolate, which were collected from chest wound swabs from patients in an adult surgical ICU ward. In addition, one *E. cloacae *isolate was obtained from central venous line of a patient in the pediatric ICU ward. Informed written consents were obtained from patients. Identification of the isolates was performed using Phoenix^® ^bacterial identification panels (NMIC/ID-107) and API^® ^20E strips (Biomerieux SA, Marcy-l'Etoile, France).

### Susceptibility test

Antibiotic susceptibility was tested using disk diffusion with the following drugs: cefotaxime, ceftazidime, tetracycline, gentamicin, amikacin, ciprofloxacin, and sulfamethoxazole. ESBL production was investigated using cefotaxime and ceftazidime, alone and in combination with clavulanic acid (BBL, Beckton Dickinson, Sparks, MD., USA) as recommended by the Clinical Laboratory Standard Institute [14]. The minimum inhibitory concentrations (MICs) of cefpodoxime, cefepime, cefoxitin, aztreonam, and imipenem, and the β-lactam/β-lactamase inhibitor combinations: cefpodoxime/clavulanate, and cefepime/clavulanate were determined by broth microdilution according to CLSI guidelines [14] using TREK microbroth dilution panels (Cleveland, Ohio, USA).

### β-lactamase characterization

Crude β-lactamase extracts from the clinical isolates and strains producing reference β-lactamases were assessed for β-lactamase pI values, inhibitor and substrate characteristics by isoelectric focusing (IEF) [15].

### β-lactamase gene identification and analysis of upstream region

PCR amplification was used to identify the presence of *bla*_CTX-M-15-like _in the *E. coli *clinical isolate, and *bla*_CTX-M-14-like _in *K. pneumoniae *and *E. cloacae *isolates using specific primers that targeted CTX-M group I and IV; respectively [16]. The presence of genes encoding TEM and SHV enzymes was analyzed by PCR [17]. The MgCl_2 _concentration used was 2 mM for *bla*_TEM _and *bla*_SHV _PCR and 1.5 mM for *bla*_CTX-M _PCR. Template DNA preparation and PCR amplifications were carried out as previously described [17].

PCR amplification and sequencing of the full-length *bla*_CTX-M-14-like _gene was performed, using primers that flanked the gene (CTXM14 F1 5'-GAG TGT TGC TCT GTG GAT AAC-3', designed using accession number AF252622 and annealing at positions 1857–1876; and CTX14R4 5'-GTT ACA GCC CTT CGG CGA TG-3' designed using accession number AF252622 and annealing at positions 2617-2598). Sequence analysis of the *bla*_CTX-M-15-like _gene was done using CTX3 FLF 5'-CGT CTC TTC CAG AAT AAG G-3', designed using accession number AY995205 and annealing at positions 169–187; and CTX3 FLR 5'-GTT TCC CCA TTC CGT TTC CGC-3' designed using accession number AY995205 and annealing at positions 1092-1072).

Sequence analysis of the 524 bp upstream region of the structural gene for *bla*_CTX-M-15 _was performed on an amplified product generated using primers IS*Ecp1 *(AGC CAA ATA CGA CAT GGC GGT G, this primer corresponds to nucleotide numbers 1179 to 1200 of the sequence with accession no. DQ658222) and CTX15 (CTT CCT AAC AAC AGC GTG AC, this primer corresponds to nucleotide numbers 261-242 of the sequence with the accession number AY995205). The 184 bases upstream of the *bla*_CTX-M-14 _were sequenced using the CTX14 upstream primer (GCA CCT GCG TAT TAT CTG C, this primer corresponds to nucleotide numbers 184-166 of the sequence with the accession number DQ359215).

Five microliters aliquots of PCR products were analyzed by gel electrophoresis with 1% agarose gels (BioRad, Hercules, Calif.) in TAE buffer. Gels were stained with ethidium bromide (10 mg/L) and visualized by UV transilluminator.

The PCR products were purified with Microcon YM-50 columns (Micon bioseparations, Bedford, MS, USA). The amplicons were sequenced using automated PCR cycle sequencing with dye terminator chemistry using ABI PRISM 3100 Genetic Analyzer and Data collection software (version 3.7).

The nucleotide and deduced amino acid sequences were analyzed and compared using BLAST software available online at http://www.ncbi.nlm.nih.gov/BLAST.

### Conjugation experiments

To determine whether the cefotaxime resistance was carried on a conjugative plasmid, conjugation experiments were performed with *K. pneumoniae *(only one isolate was tested), *E. coli *and *E. cloacae *as donors and the *E. coli *(Na azide^R^) as the recipient. The filter mating technique was carried out as previously described [18]. Transformants were selected on Mueller Hinton agar plates containing sodium azide 200 mg/L and cefotaxime 2 mg/L and were confirmed for *bla*_CTX-M _genes using PCR as described above.

## Results and discussion

### Antimicrobial susceptibility

Disk diffusion showed that all isolates were resistant to cefotaxime and positive for ESBL production by disk confirmatory test using cefotaxime/clavulanate and ceftazidime/clavulanate (Table 1). The MICs of β-lactams and β-lactam/inhibitor combinations were determined by broth microdilution technique. All clinical isolates were resistant to cefpodoxime, cefepime and resistant or intermediately resistant to aztreonam. The phenotypic ESBL microdilution confirmatory test was positive, showing a decrease by 7 doubling dilutions in the presence of clavulanic acid (Table 2). The *K. pneumoniae *clinical isolates were also resistant to other non-β-lactam antibiotics such as tetracycline, gentamicin and fluoroquinolones (Table 1).

**Table 1:** Susceptibility data of the clinical isolates and the transconjugant

	Zone diameter of inhibition (mm)								
	
Clinical Isolate	CTX	CTX/CLA	CAZ	CAZ/CLA	TET	GEN	AMK	CIP	SXT
Kp4	6	18	18	25	6	8	20	6	ND
Kp8	6	16	19	26	6	8	20	6	ND
Kp15	6	16	18	25	6	8	20	6	ND
ENT	10	20	21	25	20	17	21	30	ND
EC	9	22	15	26	19	19	20	32	6
TcEC^*a*^	11	31	20	32	25	ND	ND	ND	33
EC Na az^R^	34	32	29	29	22	23	ND	28	30

Isolates were tested for susceptibility using disk diffusion according to CLSI guidelines [14].

CTX: cefotaxime, CTX/CLA: cefotaxime/clavulanic acid, CAZ: ceftazidime, CAZ/CLA: ceftazidime/clavulanic acid, TET: tetracycline, GEN: gentamicin; AMK: amikacin; CIP: ciprofloxacin, SXT: sulfamethoxazole/trimethoprim,

TcEC: transconjugant carrying plasmid encoding *bla*_CTX-M-15_.

EC Na az^R^: recipient strain resistant to sodium azide

^*a *^Transconjugant was tested by PCR experiment for *bla*_CTX-M-15-like _using specific primers [16].

ND: Not determined

**Table 2:** MIC data of selected β-lactams and characteristics of β-lactamases produced by clinical isolates of Enterobacteriaceae from Egypt

Clinical Isolate	Enzyme characteristics^*a*^				Gene (s) detected by PCR^*b*^	MIC (mg/L) of:						
	pI	CTX hydrolysis	Inhibited by			CPD	CPD/CLA	FEP	FEP/CLA	FOX	ATM	IPM
			Clox	CLA								
Kp4	5.4	No	No	Yes	*bla*_TEM_	>128	1	>128	0.06	8	128	0.12
Kp8	6.3	No	No	Yes	-^*c*^	>128	2	>128	0.06	8	128	0.12
Kp15	7.6	No	No	Yes	*bla*_SHV_	>128	2	>128	<0.03	8	128	0.12
	8.0	Yes	No	Yes	*bla*_CTX-M-14_							
												
ENT	6.3	No	No	Yes	-^*c*^	>128	2	>128	<0.03	>16	32	0.25
	7.6	No	No	Yes	-^*e*^							
	8.0	Yes	No	Yes	*bla*_CTX-M-14_							
	8.9	No	Yes	No	-^*d*^							
												
EC	5.4	No	No	Yes	*bla*_TEM_	>128	0.25	>128	<0.03	<4	16	0.12
	6.0	No	No	Yes	-^*c*^							
	6.6	No	No	Yes	-^*c*^							
	9.0	Yes	No	Yes	*bla*_CTX-M-15_							

CPD: cefpodoxime, CPD/CLA: cefpodoxime/clavulanic acid, FEP: cefepime, FEP/CLA: cefepime/clavulanic acid, FOX: cefoxitin, ATM: aztreonam, IPM: imipenem.

^*a *^Enzyme Characteristics: pI: isoelectric point of crude β-lactamase extract preparations; CTX (0.75 mg/L) was used in the substrate-based IEF overlay technique, inhibitors used in the IEF overlay were clavulanic acid (1 mM) and cloxacillin (1 mM).

^*b *^*bla*_TEM_, *bla*_SHV _and *bla*_CTX-M _genes were detected by PCR experiments using specific primers. Only the *bla*_CTX-M _genes were sequenced using primers that flanked the full-length genes (See Methods section).

^*c *^PCR was not done to detect the gene that corresponds to the β-lactamase band on IEF gel.

^*d *^β-lactamase band focusing at pI value of 8.9, which is inhibited by cloxacillin, corresponds to the chromosomal *ampC *gene of *E. cloacae *(PCR data not shown).

^*e *^PCR was negative

Isolates of the Enterobacteriaceae producing CTX-M ESBLs are resistant to cefotaxime (MICs ≥ 64 mg/L) [9] and cefepime (MICs ≥ 32 mg/L) [19-22], but are susceptible or intermediate to ceftazidime [9]. The phenotypic characteristics of the clinical isolates in this study suggested the presence of CTX-M ESBLs. Screening using ceftazidime alone is not sufficient for organisms producing CTX-M ESBLs [16]. However, CTX-M-15 has been reported to possess some hydrolytic activity against ceftazidime [10]. The *E. coli *isolate producing CTX-M-15 was intermediate to ceftazidime using disk susceptibility test (Table 1).

### Characterization of β-lactamases

Isoelectric focusing (IEF) of crude sonicates of the clinical isolates was done by a cefotaxime/β-lactamase inhibitor overlay technique. Two enzymes focused at pI values of 8.0 and 9.0, were inhibited by clavulanic acid, and showed an extended spectrum of activity by hydrolyzing cefotaxime (Table 2). PCR and sequence analysis identified *bla*_CTX-M-14 _in one isolate of *K. pneumoniae *(KP 4) and the *E. cloacae *isolate, and *bla*_CTX-M-15 _in the *E. coli *clinical isolate (Table 2). Only one *K. pneumoniae *isolate was evaluated by sequence analysis because all three of the *K. pneumoniae *isolates showed the same enzymes on the IEF gel (Table 2).

All isolates produced multiple β-lactamases that were inhibited by clavulanate: *K. pneumoniae *(pI values 5.4, 6.3, 7.6, and 8.0), *E. cloacae *(pI values 6.3, 7.6, 8.0), and *E. coli *(pI values, 5.4, 6.0, 6.6 and 9.0) (Table 2). The *bla*_TEM _gene was detected in all *K. pneumoniae *and *E. coli *isolates, and corresponded to the β-lactamase band that focused at pI value of 5.4 when evaluated by IEF (Table 2). The *bla*_SHV _gene was present in the *K. pneumoniae *isolates (β-lactamase band focusing at pI value, 7.6-Table 2). The β-lactamase band that focused at pI value of 7.6 in the *E. cloacae *isolate was most likely not an SHV-enzyme since SHV-specific PCR was negative. Further sequencing experiments for the *bla*_TEM _and *bla*_SHV _genes were not done.

Analysis of the upstream sequence of *bla*_CTX-M-14 _and of *bla*_CTX-M-15 _revealed the presence of the right terminal inverted repeat of the insertion sequence IS*Ecp1 *and the putative promoter region (-10 and -35) associated with this element [23].

The results of the conjugation experiment showed that *bla*_CTX-M-15 _was carried on a conjugative plasmid (Table 1). The movement of *bla*_CTX-M-15 _was verified in the transconjugant using CTX-M-group 1-specific PCR [16]. The *bla*_CTX-M-14 _gene was not mobilized by conjugation.

A surveillance report on antibiotic resistance in the Southeastern Mediterranean region screened only *E. coli *isolates from different medical centers in Egypt [2]. Other important nosocomial isolates such as *K. pneumoniae *and *E. cloacae *were not evaluated in that study [2]. A recent outbreak was reported in a neonatal intensive care unit in Cairo, Egypt, in which 80% of the isolates were *K. pneumoniae*, of which 58% were ESBL producers [24]. Therefore, it is important not to limit extended-spectrum cephalosporin susceptibility screening in Egypt to *E. coli *but to include *K. pneumoniae *as well as other Enterobacteriaceae such as *E. cloacae*.

It is important for clinical microbiologists in Egyptian hospitals to screen for CTX-M ESBL producers. In addition, clinical microbiologists and physicians need to be aware that these enzymes are present in many different types of Enterobacteriaceae. This information is essential for determining the most appropriate empirical antibiotic therapy.

## Conclusion

This study is the first documentation of CTX-M-14 ESBLs in *K. pneumoniae *and *E. cloacae *isolates in Egypt as well as the Middle East region and North Africa.

## Competing interests

The authors declare that they have no competing interests.

## Authors' contributions

NK participated in the design of the study. NK carried out the susceptibility testing, molecular genetic studies, and sequence alignment; and participated in drafting the manuscript. NDH participated in the design of the study and drafting the manuscript, and coordination. Sequencing primers were designed by NDH. Work was carried out at the laboratory of Dr. Hanson, Department of Microbiology and Immunology, Creighton University, Omaha, Nebraska. All authors have analyzed and interpreted the data, and have read and approved the final manuscript.

## Pre-publication history

The pre-publication history for this paper can be accessed here:

http://www.biomedcentral.com/1471-2334/9/84/prepub

## References

[B1] El Kholy A Baseem H Hall GS Procop GW Longworth DL Antimicrobial resistance in Cairo, Egypt 1999–2000: a survey of five hospitals J Antimicrob Chemother 2003 51 3 625 630 10.1093/jac/dkg101 12615864

[B2] Borg MA Scicluna E de Kraker M Sande-Bruinsma N van de Tiemersma E Gur D Ben Redjeb S Rasslan O Elnassar Z Benbachir M Antibiotic resistance in the southeastern Mediterranean – preliminary results from the ARMed project Euro Surveill 2006 11 7 164 167 16966796

[B3] Bouchillon SK Johnson BM Hoban DJ Johnson JL Dowzicky MJ Wu DH Visalli MA Bradford PA Determining incidence of extended spectrum beta-lactamase producing Enterobacteriaceae, vancomycin-resistant Enterococcus faecium and methicillin-resistant Staphylococcus aureus in 38 centres from 17 countries: the PEARLS study 2001–2002 Int J Antimicrob Agents 2004 24 2 119 124 10.1016/j.ijantimicag.2004.01.010 15288309

[B4] Paterson DL Bonomo RA Extended-spectrum beta-lactamases: a clinical update Clin Microbiol Rev 2005 18 4 657 686 16223952 10.1128/CMR.18.4.657-686.2005 PMC1265908

[B5] Mohamed Al-Agamy MH El-Din Ashour MS Wiegand I First description of CTX-M beta-lactamase-producing clinical Escherichia coli isolates from Egypt Int J Antimicrob Agents 2006 27 6 545 548 10.1016/j.ijantimicag.2006.01.007 16713187

[B6] Moubareck C Daoud Z Hakime NI Hamze M Mangeney N Matta H Mokhbat JE Rohban R Sarkis DK Doucet-Populaire F Countrywide spread of community- and hospital-acquired extended-spectrum beta-lactamase (CTX-M-15)-producing Enterobacteriaceae in Lebanon J Clin Microbiol 2005 43 7 3309 3313 16000453 10.1128/JCM.43.7.3309-3313.2005 PMC1169093

[B7] Touati A Benallaoua S Forte D Madoux J Brasme L de Champs C First report of CTX-M-15 and CTX-M-3 beta-lactamases among clinical isolates of Enterobacteriaceae in Bejaia, Algeria Int J Antimicrob Agents 2006 27 5 397 402 10.1016/j.ijantimicag.2005.12.007 16621456

[B8] Jouini A LV Ben Slama K Sáenz Y Klibi Hammami S Boudabous A Torres Carmen Characterization of CTX-M and SHV extended-spectrum beta-lactamases and associated resistance genes in Escherichia coli strains of food samples in Tunisia J Antimicrob Chemother 2007 60 1137 1141 10.1093/jac/dkm316 17855726

[B9] Bonnet R Growing group of extended-spectrum beta-lactamases: the CTX-M enzymes Antimicrob Agents Chemother 2004 48 1 1 14 14693512 10.1128/AAC.48.1.1-14.2004 PMC310187

[B10] Poirel L Gniadkowski M Nordmann P Biochemical analysis of the ceftazidime-hydrolysing extended-spectrum beta-lactamase CTX-M-15 and of its structurally related beta-lactamase CTX-M-3 J Antimicrob Chemother 2002 50 6 1031 1034 10.1093/jac/dkf240 12461028

[B11] Poirel L Naas T Le Thomas I Karim A Bingen E Nordmann P CTX-M-type extended-spectrum beta-lactamase that hydrolyzes ceftazidime through a single amino acid substitution in the omega loop Antimicrob Agents Chemother 2001 45 12 3355 3361 11709308 10.1128/AAC.45.12.3355-3361.2001 PMC90837

[B12] Poirel L Kampfer P Nordmann P Chromosome-encoded Ambler class A beta-lactamase of Kluyvera georgiana, a probable progenitor of a subgroup of CTX-M extended-spectrum beta-lactamases Antimicrob Agents Chemother 2002 46 12 4038 4040 12435721 10.1128/AAC.46.12.4038-4040.2002 PMC132763

[B13] Humeniuk C Arlet G Gautier V Grimont P Labia R Philippon A Beta-lactamases of Kluyvera ascorbata, probable progenitors of some plasmid-encoded CTX-M types Antimicrob Agents Chemother 2002 46 9 3045 3049 12183268 10.1128/AAC.46.9.3045-3049.2002 PMC127423

[B14] CLSI Performance Standards for Antimicrobial Disk Susceptibility Tests-Ninth Edition: Approved Standard M-2-A9 2006 Clinical Laboratory Standards Institute (CLSI), Wayne, PA, USA

[B15] Sanders CC Sanders WE Jr Moland ES Characterization of beta-lactamases in situ on polyacrylamide gels Antimicrob Agents Chemother 1986 30 6 951 952 3492960 10.1128/aac.30.6.951 PMC180628

[B16] Pitout JD Hossain A Hanson ND Phenotypic and molecular detection of CTX-M-beta-lactamases produced by Escherichia coli and Klebsiella spp J Clin Microbiol 2004 42 12 5715 5721 15583304 10.1128/JCM.42.12.5715-5721.2004 PMC535227

[B17] Hanson ND Thomson KS Moland ES Sanders CC Berthold G Penn RG Molecular characterization of a multiply resistant Klebsiella pneumoniae encoding ESBLs and a plasmid-mediated AmpC J Antimicrob Chemother 1999 44 3 377 380 10.1093/jac/44.3.377 10511405

[B18] Pitout JD Thomson KS Hanson ND Ehrhardt AF Coudron P Sanders CC Plasmid-mediated resistance to expanded-spectrum cephalosporins among Enterobacter aerogenes strains Antimicrob Agents Chemother 1998 42 3 596 600 9517938 10.1128/aac.42.3.596 PMC105504

[B19] Yu WL Pfaller MA Winokur PL Jones RN Cefepime MIC as a predictor of the extended-spectrum beta-lactamase type in Klebsiella pneumoniae, Taiwan Emerg Infect Dis 2002 8 5 522 524 11996691 10.3201/eid0805.010346 PMC2732501

[B20] Moland ES Black JA Hossain A Hanson ND Thomson KS Pottumarthy S Discovery of CTX-M-like extended-spectrum beta-lactamases in Escherichia coli isolates from five US States Antimicrob Agents Chemother 2003 47 7 2382 2383 12821506 10.1128/AAC.47.7.2382-2383.2003 PMC161829

[B21] Bouallegue-Godet O Ben Salem Y Fabre L Demartin M Grimont PAD Mzoughi R Weill FX Nosocomial Outbreak Caused by Salmonella enterica Serotype Livingstone Producing CTX-M-27 Extended-Spectrum beta-Lactamase in a Neonatal Unit in Sousse, Tunisia J Clin Microbiol 2005 43 3 1037 1044 15750057 10.1128/JCM.43.3.1037-1044.2005 PMC1081247

[B22] Schlesinger J Navon-Venezia S Chmelnitsky I Hammer-Munz O Leavitt A Gold HS Schwaber MJ Carmeli Y Extended-spectrum beta-lactamases among Enterobacter isolates obtained in Tel Aviv, Israel Antimicrob Agents Chemother 2005 49 3 1150 1156 15728917 10.1128/AAC.49.3.1150-1156.2005 PMC549242

[B23] Poirel L Decousser JW Nordmann P Insertion sequence ISEcp1B is involved in expression and mobilization of a bla(CTX-M) beta-lactamase gene Antimicrob Agents Chemother 2003 47 9 2938 2945 12936998 10.1128/AAC.47.9.2938-2945.2003 PMC182628

[B24] Moore KL Kainer MA Badrawi N Afifi S Wasfy M Bashir M Jarvis WR Graham TW el-Kholy A Gipson R Neonatal sepsis in Egypt associated with bacterial contamination of glucose-containing intravenous fluids Pediatr Infect Dis J 2005 24 7 590 594 10.1097/01.inf.0000168804.09875.95 15998998

